# Clinical application of optical coherence tomography for the imaging of non–melanocytic cutaneous tumors: a pilot multi–modal study

**Published:** 2010-11-25

**Authors:** AM Forsea, EM Carstea, L Ghervase, C Giurcaneanu, G Pavelescu

**Affiliations:** * Dermatology Department Elias University Hospital, Carol Davila University of Medicine and Pharmacy, Bucharest Romania; ** National Institute for Optoelectronics INOE 2000, MagureleRomania

**Keywords:** skin cancer, skin imaging, optical coherence tomography, non–invasive diagnosis

## Abstract

Context: Optical coherence tomography (OCT) is an emergent imaging technique, based on the interference of infrared radiation and living tissues, that allows the in vivo visualisation of the skin structures, at high resolution and up to 1.6 mm depth. As such, there is mounting evidence that OCT may be an interesting technique for the diagnossis of skin diseases, including the non–invasive early detection of cutaneous tumors.

Objective: We aimed to investigate the utility of OCT for the diagnosis of non–melanocytic, non–pigmented cutaneous tumors.

Methods: Preliminary results are presented from an initiated study. Fifteen consecutive patients with clinical suspicion of epithelial cancers and precancers registered over one week in an university dermatologic department were included. As control were selected 7 patients with inflammatory skin diseases (psoriasis, lichen planus, cutaneous lupus erythematosus). In all study and control patients the lesions and samples of normal, perilesional skin were documented by clinical digital photography, contact dermoscopy with digital image capture and OCT with central wavelength of 930 nm. Final diagnosis was certified by histopathological analysis.

Results: We could identify morphological features in OCT examination that distinguished between normal and lesional skin, and between neoplastic vs. inflamatory lesions. In the same time, combining OCT and dermatoscopical evaluation of a lesion improved the performance of diagnosis when compared to clinical diagnosis alone and with either OCT or dermoscopy imaging used alone.

Conclusions: OCT appears as a promising method of  in vivo diagnosis of early neoplastic cutaneous lesions with equivocal clinical and/or dermoscopic aspect. Continuation of our study as well as other larger investigation will be able to contribute with new insights in the role of OCT in the non–invasive diagnosis of skin disease.

## Introduction

**Figure 1:**
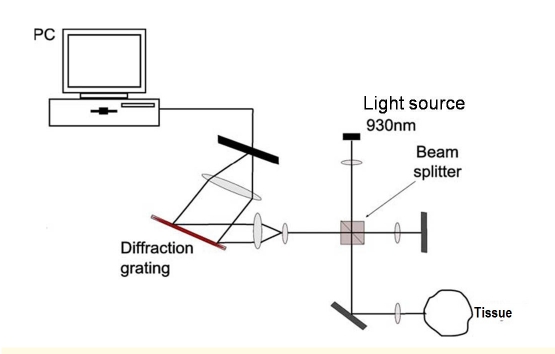
Principle of function of Optical coherence

Optical coherence tomography (OCT) is an emergent in vivo imaging technique, based on the interference of infrared radiation (900–1500nm) and living tissues, that allows the non–invasive, high resolution, two– or three–dimensional, cross–sectional visualisation of microstructural morphology of skin structures [1]. OCT is based on the principle of Michelson interferometry [2]. The device sends a light beam to the tissue to be analyzed and records the signals generated by the interference of the light reflected from the tissue with a reference beam (Figure 1).

Measurement of the interference pattern allows to determine the position of different absorbent or reflective tissues components, such as cell membranes or melanin which provide the contrast in the images [3]. This technique was initially developed in medical practice in the field of ophthalmology [4] and cardio–vascular surgery and consequently found its use as diagnosis procedure in different medical disciplines. The studies using OCT to analyze skin structure in clinical setting started in the last decade [5] and consequently proved that this technique is useful in visualizing subsurface structures of normal skin, including the dermo–epidermal junction, hair follicles, blood vessels, and sweat ducts [6–8]. 

Recently an increasing number of publications brought evidence of the utility and the precision of this technology, in its different technical variants, in diagnosing skin lesions, including malignancies[9–15].  Because this technique allows direct imaging of superficial tissue morphology at depth of up to 2 mm, at a resolution equivalent to a low–power microscope, it represents a promising tool for non–invasive evaluation of early, superficial, pre–malignant and malignant lesions, especially for their early detection and treatment follow–up. Still, the encouraging results regarding the use of OCT in dermatology come from recent reports of case presentations and small clinical studies, with limited statistical significance.  Up to present there are no definitive morphologic diagnostic criteria for OCT in skin pathology and intensive research efforts are dedicated to correlate the cutaneous morphology from OCT with the histopathological aspects and with the morphological features obtained with other validated or developing imaging methods, like dermoscopy or confocal laser microscopy (CFLM). In this context we aimed to investigate, in national premiere, the utility of OCT for the diagnosis of non–melanocytic, malignant lesions in clinical setting.

## Methods|materials

### Subjects

We present here preliminary results from a larger initiated prospective study, aimed to characterize the OCT morphological aspects of non–melanocytic premalignant and malignant lesions in clinical setting. 15 consecutive patients who presented with clinical suspicion of early epithelial cancers and precancers over one week in a university dermatologic department were included in the study after informed consent. Large, exophytic or ulcerated tumors were excluded. As control we selected 5 patients presented in the same time interval with non–malignant skin diseases, but with clinical aspect included in the differential diagnosis of early epithelial neoplasia, i.e. erythematous, squamous patch/nodules. In both study and control patients the lesions and samples of normal, perilesional skin were documented by clinical digital photography, contact dermoscopy with digital image capture and OCT. All final diagnoses were confirmed by histopathological analysis in standard haematoxyllin–eosin stain.

### OCT measurements 

OCT measurements were performed with a Thorlabs OCP930SR Spectral Radar OCT device, equipped with a 930 nm light source, of 2mW optical power. This scanning device has a spectral bandwidth of 100 nm, yielding a typical imaging depth of aprox. 1.6 mm, 20 micro m lateral resolution and a 6.2  micro m axial resolution. Images were acquired at 8 fps, maximum image width of 6 mm and image size of 512 rows. The duration of the scan acquisition was 5 to 10 seconds. The scans were performed through the mid part of the largest diameter of the lesions. In order to optimize optical coupling of the probe with the uneven and mobile surface of the skin, we used ultrasound gel as a contact medium applied to the skin. This provided the best image quality compared with other topical agents that we tried (mineral oil, paraffin, alcohol and vaseline). The ultrasound gel layer was visible through all our images as a bright line at skin surface.

## Results 

The study lot of 15 patients had the final diagnoses, histopathologically confirmed, of 3 basal cell carcinoma, 1 keratoacanthoma, 4 actinic keratoses, 1 Bowen disease, 2 sebaceous hyperplasia, 1 Kaposi’s angio–sarcoma, 1 sebaceous cyst and 2 dermatofibromas. Six lesions could not be evaluated by OCT, because of too high elevation of the lesion, patient's movement or unfavorable location. Within the control lot, 3 patients were diagnosed with psoriasis, 1 with cutaneous lupus erythematosus, 1 with cutaneous sarcoidosis, and for comparison we analyzed also 2 seborrheic keratoses.

### Normal skin structures 

**Figure 2:**
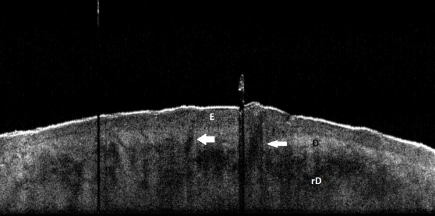
Normal skin, OCT aspect.  Extensor aspect forearm, 53 year old male; E epidermis, D Dermis, rD reticular dermis, white arrows: hair follicles

Our preliminary results demonstrated that OCT was useful in visualizing and differentiating skin layers and structures such as epidermis, papillary and reticular dermis, dermal blood vessels, hair follicles and glandular ducts (Figure 2–Figure 4).

It also distinguished the thickness and structure variations according to different skin types, anatomical location and age. Generally, the epidermis appeared darker as the underlying dermis, with dermo–epidermal junction clearly visible in most images. Dark, signal–poor, round or thin branched spaces appeared in the dermis, corresponding to different–sized blood vessels. Stratum corneum was visible as a thin bright line. Reticular dermis structures were usually effaced. 

**Figure 3:**
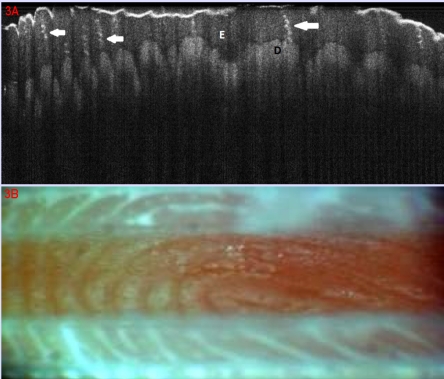
Normal skin. Volar aspect of index finger; (A). OCT aspect: E epidermis, D dermis (dermal papillae), white arrows: sweat ducts; (B). Clinical aspect of dermatoglyphs

**Figure 4:**
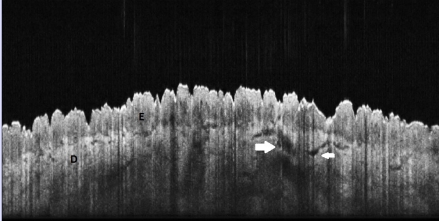
Normal skin, OCT aspect. Thigh, 53 years old female, E: epidermis, D: dermis, white arrows: blood vessels

### Actinic keratoses and Bowen's disease 

**Figure 5:**
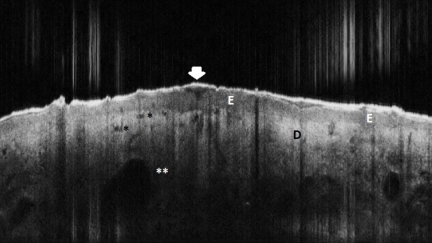
Actinic Keratosis, OCT aspect. E: epidermis, slightly thickened in the lesional area; Arrowhead: signal–poor irregular band corresponding to hyperkeratosis; D dermis; asterisc black: round signal–free structures corresponding to dilated blood vessels; double asterisc white: venectasies

**Figure 6:**
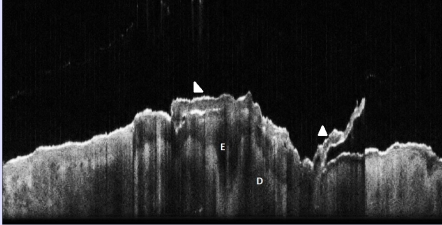
Bowen's Disease, OCT aspect. E: epidermis, which in the lesional area is markedly thickened, irregular, hypo–dense; one hair follicle appears to be involved;  Hyperkeratosis is also marked,  with a scale (arrowhead) partially detached. There is marked signal attenuation in the dermis (D)

The actinic keratoses and the in situ squamous cell carcinoma (Figure 5–Figure 6) showed various degrees of epidermal thickening, with lesional epidermis appearing darker, possibly due to increased keratin content. An overlying dark irregular band corresponding to hyperkeratosis was present. The lateral limits of the lesions were visible as well as the dermal–epidermal demarcation. Within the in situ squamous cell carcinoma (Figure 6) the marked increased thickness of epidermis is highly irregular and scales appearing bright are visible.

### Basal cell carcinoma

**Figure 7:**
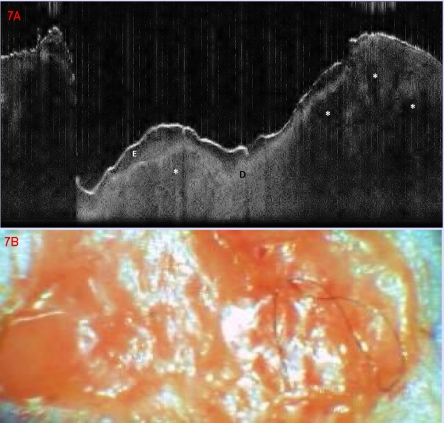
Basal cell carcinoma. (A) OCT aspect, ; lobular signal–poor structure, corresponding to tumoral lobules; E epidermis, D Upper dermis; Upper dark band corresponds to keratin–rich stratum corneum; (B) Clinical aspect

**Figure 8:**
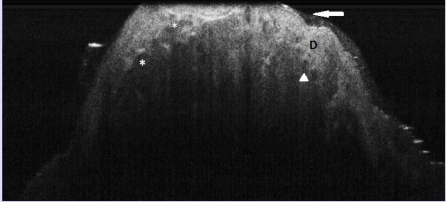
Basal cell carcinoma, nodular type, OCT aspect. OCT aspect: ; signal–poor lobulated structures corresponding to tumoral lobules; D upper dermis; arrow: thin epidermis, difficult to distinguish from underlying dermis; arrowhead: round signal–free structures corresponding to cross–section blood vessels

The basal cell carcinomas that we evaluated through OCT (Figure 7–Figure 8) showed characteristic lobular signal–poor structures which occupied the dermis and corresponded on the histopathology examinations to tumoral lobules. One tumor depicted (Figure 7) was a superficial BCC showing ulceration, the other (Figure 8) was nodular of solid type. Signal attenuation in the dermis in OCT did not allow proper assessment of the tumor limits in depth, beyond 1 mm.

### Kaposi sarcoma

**Figure 9:**
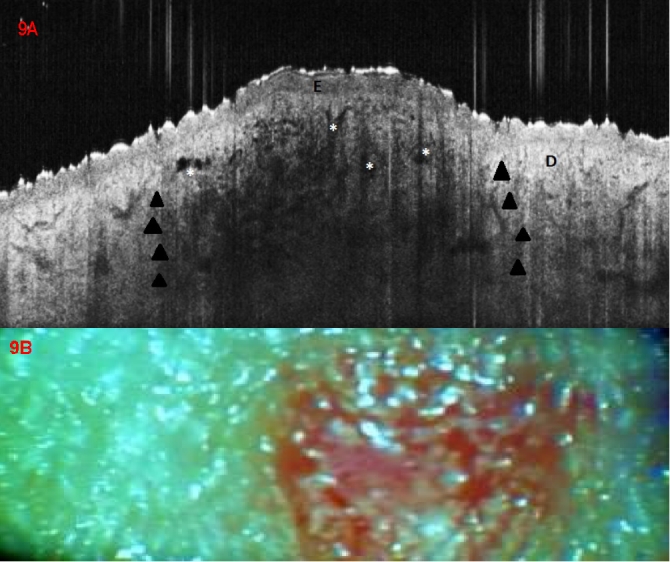
Kaposi Sarcoma. (A) OCT aspect, focal changes in the central lesional area, showing a flattened epidermis (E), irregular, partly round partly branched and reticular signal–ppoor/free structures (asterisc) corresponding to vascular spaces, and a nodular area of attenuated signal (dark arrowheads) in the dermis (D); (B) clinical aspect of a small, superficial, red-bluish nodule on the knee of a 64 years old male

Interestingly, in one patient with early lesions of Kaposi's sarcoma OCT scanning showed a nodular area of signal attenuation in the dermis, corresponding to histopathological and clinical aspect of a small incipient angio–sarcomatous nodule (Figure 9). Within it, branched, reticular or round signal–poor/free structures were visible, corresponding to vascular spaces and clearly demarcated from the unaffected perilesional dermis. The epidermis was distinguishable from underlying dermis, with flattening over the lesion.

### Seborrheic keratoses

**Figure 10:**
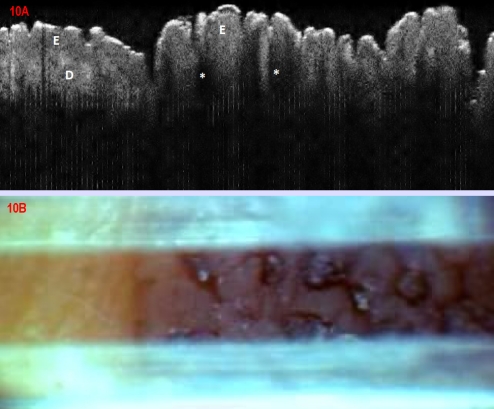
Seborrhoic keratosis. (A) OCT aspect E: epidermis, which in the lesional area, on the right half of the image shows marked thickening, with papillomatous aspect, as well as rounded hypo–dense structures (asteriscs), possibly corresponding to horn pseudocysts; marked signal attenuation over the dermis (D). (B) Clinical aspect of the margin area of a seborrhoic keratosis on the trunk

Two seborrheic keratoses, with typical clinical aspect were included as control lesions in the study. On OCT they showed characteristical changes (Figure 10) as compared to normal skin, with marked thickening of the epidermis with papillomatous aspect, as well as superficial rounded hypo–dense structures, unsharply demarcated, which corresponded to horn pseudocysts; these latter structures were easy to differentiate from the more uniformly dark, and sharp–demarcated round/oval ones corresponding to vessels visualized in OCT. Increased thickness of epidermis was associated with marked signal attenuation over the dermis.

### Inflammatory skin lesions

**Figure 11:**
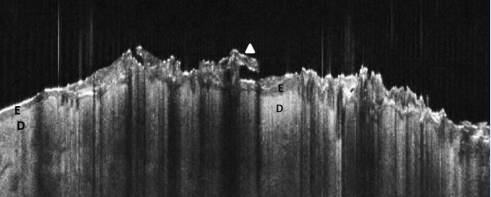
Psoriasis, OCT aspect. Epidermis (E) is hypo–dense and in the lesional area (left) is markedly thickened, with elongated protrusions in underlying dermis (D); overlying marked focal hyperkeratosis, as dark band, with scales protruding (arrowhead). There are longitudinal bands of signal attenuation corresponding to scaling

**Figure 12:**
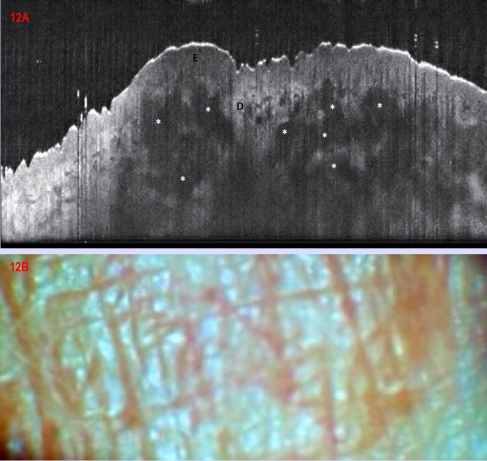
Cutaneous  Sarcoidosis.(A) OCT aspect showing signal–poor agglomerated masses (asteriscs) occupying the dermis in the lesional area ( left), corresponding to granulomatous infiltrates. E epidermis; D dermis unaltered in the right extreme of the image, showing vascular spaces; (B) Clinical aspect of a well delimitated erythematous–brownish patch on the abdomen

The control lesions we included in the study comprised 3 psoriasis lesions (Figure 11), which in OCT showed typical thickening of the epidermis, with protrusions in the dermis, strong hyperkeratosis as a darker superficial band and signal–poor rounded structures in the dermis corresponding to dilated vessels. Another control case of sarcoidosis showed in OCT hypo–dense confluent rounded masses occupying the dermis, and corresponding on histopathology exam to granulomatous infiltrates (Figure 12).

## Discussion

Our preliminary results showed that OCT is a valuable technique of non–invasive visualisation of skin structures and texture variations. They also demonstrated several OCT features of early epithelial cancers and precancers of the skin that could be visualized and correlated with standard histopathology. It was possible to differentiate, by means of OCT between normal and lesional skin, to demonstrate abnormal skin architecture in most of the investigated lesions along with subtle differences of signal–transducing properties of the skin layers reflecting modified cytologic characteristics, as well as to visualize the lateral delimitations of lesions. This technique was particularly useful to differentiate squamous cell dysplastic lesions from superficial basal cell carcinomas, and to suggest diagnosis for early Kaposi's sarcoma and sarcoidosis respectively, significantly adding useful information to the clinical inspection and dermoscopy examination of these lesions. 

Our results are in accord with very recent reports that showed a high diagnostic accuracy of OCT in differentiating normal skin from cutaneous lesions, with sensitivity of 79%–94%, and specificity of 85%–96%, while the diagnosis performance of a particular type of early neoplastic lesions varied greatly, with sensitivities between 60% to 100% and specificities between 60% and 91𠀕 for basal cell carcinoma or squamous cell carcinoma[12,14,16]. Most of the recent studies identified characteristic OCT morphological aspects, allowing to detect neoplastic cutaneous lesions. However, specific and systematic studies of diagnostic sensitivity and specificity, at larger scale that would allow statistical significance are still lacking, while the correlation between OCT skin morphology and histopathology is still under study. 

The general limitations of the OCT method consist in the impossibility to obtain important information on the cellular level at the resolution used in the present study, as well as the limited penetration depth and the size of sampled field. The development of high–output broadband light sources and functional OCT imaging based on spectroscopy, tissue birefringence, and Doppler flow[17–18] represent important steps toward the solution of these problems, as well as polarization–sensitive OCT (PS–OCT) [19] or speckle–reduced OCT [20] may increase the diagnostic accuracy. A further area of expected progress is the continuous development of image analysis, machine learning algorithms, and neural networks [21] which would surpass the diagnostic capacities of human eye. 

Our study was also limited by the small number of cases of different cutaneous pathologies presented. These represent the preliminary encouraging results of a larger prospective study that we initiated to analyze the OCT morphology in early neoplastic skin lesions.

## Conclusions 

Optical coherence tomography appears to be a promising method of non–invasive diagnosis and treatment monitoring of early neoplastic cutaneous lesions with equivocal clinical and/or dermoscopic aspect. OCT images showed good diagnostic accuracy, allowing to differentiate normal skin layers and structures from cutaneous lesions. It also distinguished the thickness and structure variations according to different skin types, anatomical location and age.

All acquired OCT results were confirmed by the histopathological and clinico–morphological examination. Seborrheic keratoses, with papillomatous aspect, and inflammatory skin diseases, were used as control data. Actinic keratoses and the in situ squamous cell carcinoma were characterized by irregular epidermal thickening and hyperkeratosis, while basal cell carcinoma showed lobular tumoral structures in the dermis and Kaposi sarcoma was evidenced by a nodular, inhomogenous area of signal attenuation in the dermis with vascular–like signal–poor structures. Combination of OCT with other imaging techniques such as dermoscopy or confocal laser microscopy may significantly improve the early detection as well as the monitoring of conservative treatment of these lesions. Continuation of our study as well as other larger clinical trials will contribute with new insights and with the necessary evidence–based data to clarify the role of OCT and its progressive variants in the non–invasive diagnosis of skin diseases.
